# *Sodalis glossinidius* prevalence and trypanosome presence in tsetse from Luambe National Park, Zambia

**DOI:** 10.1186/1756-3305-7-378

**Published:** 2014-08-19

**Authors:** Jonny W Dennis, Simon M Durkin, Jemima E Horsley Downie, Louise C Hamill, Neil E Anderson, Ewan T MacLeod

**Affiliations:** Division of Pathway Medicine, School of Biomedical Sciences, College of Medicine and Veterinary Medicine, The University of Edinburgh, Chancellor’s Building, 49 Little France Crescent, Edinburgh, EH16 4SB UK; The Royal (Dick) School of Veterinary Studies, University of Edinburgh, Roslin, EH25 9RG UK

**Keywords:** *Glossina*, *Rhodesiense*, Wildlife, *Morsitans*, *Pallidipes*, *Brevipalpis*

## Abstract

**Background:**

Tsetse flies are the biological vectors of African trypanosomes, the causative agents of sleeping sickness in humans and nagana in animals. The tsetse endosymbiont *Sodalis glossinidius* has been suggested to play a role in tsetse susceptibility to infection. Here we investigate the prevalence of African trypanosomes within tsetse from the Luambe National Park, Zambia and if there is an association between *S. glossinidius* and presence of trypanosomes within the tsetse examined.

**Methods:**

Tsetse representing three species (*Glossina brevipalpis*, *Glossina morsitans morsitans* and *Glossina pallidipes*), were sampled from Luambe National Park, Zambia. Following DNA extraction, PCR was used to examine the tsetse for presence of trypanosomes and the secondary endosymbiont *S. glossinidius*.

**Results:**

*S. glossinidius* infection rates varied significantly between tsetse species, with *G. brevipalpis* (93.7%) showing the highest levels of infection followed by *G. m. morsitans* (17.5%) and *G. pallidipes* (1.4%). ITS-PCR detected a wide variety of trypanosomes within the tsetse that were analysed. Significant differences were found in terms of trypanosome presence between the three tsetse species. A high proportion of *G. m. morsitans* were shown to carry *T. brucei* s.l. DNA (73.7%) and of these around 50% were positive for *Trypanosoma brucei rhodesiense. T. vivax*, *T. godfreyi*, *T. simiae*, *T. simiae* Tsavo and *T. congolense* were also detected. No association was found between the occurrence of *S. glossinidius* and the presence of trypanosome DNA in any of the three tsetse species tested.

**Conclusion:**

The current work shows that *T. b. rhodesiense* was circulating in Luambe National Park, representing a risk for people living in the park or surrounding area and for tourists visiting the park. The differences in trypanosome DNA presence observed between the different tsetse species tested may indicate host feeding preferences, as the PCR will not discriminate between a fly with an active/resident infection compared to a refractory fly that has fed on an infected animal. This makes it difficult to establish if *S. glossinidius* may play a role in the susceptibility of tsetse flies to trypanosome infection.

## Background

Tsetse flies are the biological vector of African trypanosomes, the causative agents of sleeping sickness in humans and nagana in animals. They host three symbiotic bacteria, *Wigglesworthia glossinidia*, *Sodalis glossinidius* and *Wolbachia* [1]. *W. glossinidia* is the primary symbiont and is found in all tsetse flies, its main role is in the provision of B vitamins that are absent in the tsetse diet of vertebrate blood [2]. The other two bacteria are generally found in laboratory tsetse, however, their prevalence within wild tsetse populations is variable. *S. glossinidius*, originally classified as a rickettsia-like organism [3], has no clear role within the tsetse but is thought to play a part in susceptibility to trypanosome infection [4–6]. *Wolbachia* has recently been shown to be involved in cytoplasmic incompatibility in tsetse [7].

There have been many studies over the years investigating natural rates of trypanosome infection in tsetse flies. In the past, microscopy was used to assess infection. Flies were commonly dissected using the method described by Lloyd and Johnson [8], where infection was ascertained by position of trypanosomes in the tsetse. However, infection rates in wild flies were often low, for instance Okoth and Kapaata [9] found less than 1% of flies from Uganda with a salivary gland infection. More recently PCR has been used to examine tsetse flies for trypanosome infections. This has the advantage of being able to identify trypanosomes to species/sub species level and also identify mixed infections [10]. However, PCR may over estimate tsetse infection rates, as it will not discriminate between an active infection of the fly or simply trypanosomes that have been imbibed with a bloodmeal [11].

Just over thirty years ago, Maudlin [12] established that tsetse susceptibility to midgut infection by trypanosomes was a maternally inherited characteristic. Later work associated susceptibility with the presence of *S. glossinidius* and it was suggested that the action of a chitinase, produced by *S. glossinidius*, broke down chitin during the pupation period, causing the build up of *N*-acetyl-D-glucosamine [4]. This compound was thought to inhibit tsetse lectins that killed incoming trypanosomes when tsetse fed on an infected host. More recently alternative trypanocidal mechanisms have been put forward including antimicrobial peptides [13] or oxidative stress [14]. A role for *W. glossinidia* in tsetse immunity has also been suggested, where older flies devoid of the primary symbiont show greater susceptibility to trypanosome infection than flies with the symbiont [15].

Although there have been several field studies of tsetse flies over the past thirty years looking at trypanosome infections, very little is known about the prevalence of secondary symbionts within tsetse, with very few species being investigated. Several techniques have been used to screen tsetse flies for *S. glossinidius* infection, these include electron microscopy [16, 17], dot blots with radioactive probes [18, 19] and more recently PCR [20–23]. In studies using electron microscopy [16] and PCR [21, 24], all laboratory tsetse tested positive for *S. glossinidius*. A selective advantage has been suggested for *S. glossinidius* presence based on increased puparial survival in laboratory tsetse [25]. *G. m. morsitans* from the University of Bristol colony showed a prevalence of 80% for *S. glossinidius* while samples taken from Zimbabwe, where these flies originated showed a prevalence of 20% [25].

One of the first studies to investigate the prevalence of *S. glossinidius* in wild tsetse flies was undertaken in Liberia [19]. Using radioactive dot blots to diagnose infection, *S. glossinidius* prevalence was found to be 85% (95% CI = 66.3-95.8%), 31% (95% CI = 11.0%-58.7%) and 9.3% (95% CI = 6.9%-12.2%) in *Glossina nigrofusca, Glossina pallicera* and *Glossina palpalis palpalis* respectively. An association was shown between *S. glossinidius* presence and trypanosome infection (diagnosed by microscopy) in *G. p. palpalis* with symbiont positive flies six times more likely to be trypanosome positive. In more recent years several authors have tried to show an association between *S. glossinidius* presence in wild tsetse flies and trypanosome presence using molecular techniques. Farikou *et al*. [22] showed a link between the presence of *S. glossinidius* and trypanosome infection in wild flies in Cameroon. PCR analysis showed that 54.9% (95% CI = 50.2%-59.6%) of flies tested were positive for *S. glossinidius* infection, of these 75% were also positive for trypanosome infection. This association between trypanosome susceptibility and *S. glossinidus* was also shown in *Glossina pallidipes* in Kenya, however, there were very few flies which were both *S. glossinidius* and trypanosome infected so no definitive conclusion could be made [23].

In the current work we have used PCR to detect *S. glossinidius* and an ITS-PCR [26] to detect trypanosomes in tsetse from Zambia. The latter primer set detects the following trypanosomes, *Trypanozoon* (*T. brucei brucei. T. brucei gambiense*, *T. brucei rhodesiense T. evansi* and *T. equiperdum*), *T. congolense* savannah, *T. congolense* forest, *T. congolense* kilifi, *T. simiae*, *T. simiae* Tsavo, *T. godfreyi* (all *Nannomonas*) and *T. vivax* (*Duttonella*). The majority of these trypanosomes establish within the midgut of the tsetse before moving to either the salivary glands or proboscis where they mature into the mammalian infective stage. The exceptions are *T. vivax* where the lifecycle is restricted to the mouthparts of the fly, *T. evansi,* which is mechanically transmitted and *T. equiperdum*, which is sexually transmitted.

## Methods

### Tsetse fly collection

Tsetse flies were surveyed using a stratified random sampling design. Using a land cover classification of Luambe National Park, Zambia and the surrounding area [27], five vegetation types considered the most suitable tsetse habitat were identified (riverine woodland, thicket, mopane woodland, mopane scrub woodland and *Combretum-Terminalia* woodland). A 500 m grid was placed over the study area and squares selected with a percentage composition of 55% or above for the selected vegetation classes. For mopane scrub woodland and riverine woodland lower thresholds of 50% and 10% respectively were used due to lack of larger areas of homogenous woodland for these classes. Ten trap sites, two for each vegetation type, were then randomly selected from the candidate list using a random number generator. The central point for the selected 500 m square was used to locate the trap site on the ground and assess its suitability. One trap was placed in the most suitable location as close as possible to the central point for each grid square. A second trap was then placed 200 m from the first in a direction that was perpendicular to the prevailing wind direction so that both traps were within the same grid square.

Standard Epsilon traps (Bonar Industries (Pvt) Ltd, Zimbabwe) were used to sample the tsetse flies. Traps were set once daily at 0600 and flies collected the following day at the same time. Traps were generally set without odour baits, but baits were used on each trap for a period during the study (two sachets (5 cm × 5 cm, 150 μm thick) containing 3-n-propylphenol, octenol and 4 methylphenol in the ratio 1:6:12 and an open 500 ml bottle containing methylethylketone (MEK) at the entrance to the traps). The study ran from June to October, 2006 with sampling occurring during one week of each month during that period.

During October, when ambient temperatures were at their greatest, three artificial refuges were created to sample *G. brevipalpis*. They were sited in well-shaded areas of riverine woodland within 50 m of the Luangwa River. A 200 litre metal drum with one open end was spilt in half longitudinally and buried in soil and leaf litter as described by Vale [28]. Flies were sampled over several days towards the end of the study period by placing a net over the open end of the refuge during the hottest period of the day. The tsetse sampled consisted of 419 *G. pallidipes* (*Combretum-Terminalia* woodland: 67; mopane scrub: 72; mopane woodland: 73; artificial refuge: 44; riverine woodland: 74), 137 *G. m. morsitans* (*Combretum-Terminalia* woodland: 38; mopane scrub: 28; mopane woodland: 29; riverine woodland: 21; thicket: 21) and 55 *G. brevipalpis* (artificial refuge: 54; riverine woodland: 1). Flies were stored individually in acetone and transported to Edinburgh University where they were stored at -20°C.

### DNA extraction and PCR

Frozen tsetse flies were thawed at room temperature. Flies were washed using 5% sodium hypochlorite and phosphate buffered saline (g/l NaCl 8.0, KCl 0.2, Na_2_HPO_4_ 1.15, KH_2_PO_4_ 0.2, pH 7.3) (OXOID) to remove any external sources of DNA. Flies were then crushed with pestles in individual 1.5 ml microcentrifuge tubes; pestles were thoroughly cleaned with bleach and then autoclaved between uses to prevent contamination. DNA was extracted from the tissue using the QIAGEN® DNeasy® blood and tissue kit following the manufacture’s instructions.

To test the integrity of the samples, all DNA extracts were tested for amplification in 25 μl reactions using 1 μl of template DNA for *W. glossinidia* DNA using primers targeting the 16S [29] or *fliC* gene [30]. Three *G. pallidipes* failed to show amplification for *W. glossinidia* DNA and were removed from the study set. All other tsetse extracts showed positive amplification for *W. glossinidia* and were subjected to PCR for *S. glossinidius*, targeting the *GroEL* gene [31] and African trypanosomes targeting the ITS region, which is present at around 200 copies per cell [26]. For the latter, the species of trypanosome was determined by the size of the band produced, as described by Njiru *et al*. [26]. Flies testing positive for *T. brucei* s.l. DNA using the ITS-PCR were further evaluated to examine if they were *T. b. rhodesiense* by using the PLC-SRA-PCR described by Picozzi *et al*. [32]. This multiplex PCR tests for both the single copy *SRA* gene (found in *T. b. rhodesiense*) and the single copy *PLC* gene (common to all *Trypanozoon*). If a sample is SRA positive then it is diagnosed as *T. b. rhodesiense*, a fly positive for PLC but not SRA suggests that the sample is not *T. b. rhodesiense*, however, there is enough DNA to amplify a single copy trypanosome gene if it were present. Where there is no amplification (either PLC or SRA) then there is not enough DNA to amplify a single copy gene, as such it is not possible to diagnose the infection, in this case these flies were discounted when determining the prevalence of *T. b. rhodesiense*. On occasion an artefact will occur that is indicative of a VSG gene, this occurrence will not speciate the trypanosome infection, but is indicative of the presence of parasitic material. Therefore, flies that returned a VSG gene amplification in isolation were discounted from further calculations regarding the prevalence of *T. b. rhodesiense*.

All PCRs were repeated at least twice, and in all PCRs a positive control (DNA known to test positive for that specific PCR) and a negative control (distilled water) were run. A DNA engine DYAD™ Peltier thermal cycler was used to run the PCRs, and PCR products were separated by electrophoresis on 1.5% molecular grade agarose (BIOLINE) gel stained with 0.04_ μl/ml of GelRed nucleic acid stain (BIOTIUM). Separated products were then viewed under ultraviolet light in a transilluminator (BIO-RAD), and band sizes analysed compared to exACTGene low range plus DNA ladder (Fisher Scientific International Inc.). No *T. congolense* kilifi was detected, and as it was not possible to differentiate between *T. congolense* savannah and *T. congolense* forest, any band of 700 bp was classified as *T. congolense*.

### Statistical analysis

Fisher’s exact test was used for all analyses in the study. Firstly, the prevalence of *S. glossinidius* was examined for differences between the three species of tsetse studied in the current work. Secondly, the data was examined to assess if detection of *S. glossinidius* was associated with trypanosome presence. Finally, the prevalence of trypanosomes was examined to look for statistical differences between and within tsetse species. As *T. vivax* completes its lifecycle exclusively within the proboscis of the fly it was excluded from the analysis. All analysis was carried out in WinPepi (version 11.32) and statistical significance was accepted at the 95% confidence level throughout.

### Ethical statement

All activities in protected areas were fully approved by the Zambian Wildlife Authority (permit numbers 316295 and 323947) as part of a wider study investigating trypanosomes in the Luangwa Valley [33].

## Results

### Prevalence of *S. glossinidius*in wild fly populations from Zambia

**Figure 1:**
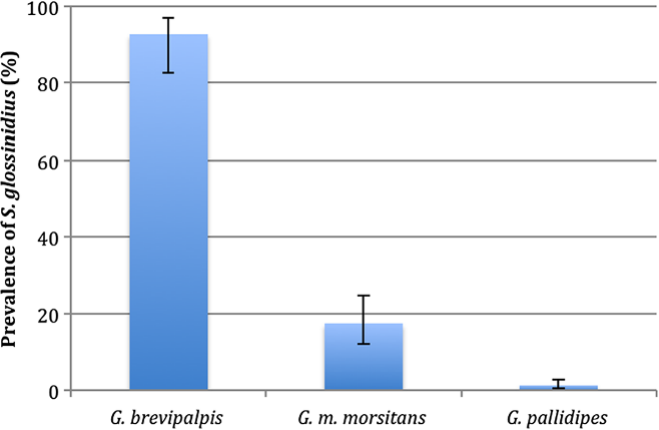
**Prevalence of** ***S. glossinidius*** **in three species of tsetse from Luambe National Park in Zambia using PCR.** The prevalence was significantly lower in *G. pallidipes* (1.4% 95% CI 0.8-1.5, n = 419) when compared to *G. m. morsitans* (17.5% 95% CI 12.1-24.7, n = 137) and *G. brevipalpis* (93.7% 95% CI 82.7-97.1, n = 55). The prevalence in *G. m. morsitans* was significantly lower than in *G. brevipalpis*. Error bars show 95% confidence intervals.

The prevalence of *S. glossinidius* in the various species of tsetse is shown in Figure 1. The prevalence of *S. glossinidius* in *G. brevipalpis* was significantly higher than in *G. m. morsitans* (p < 0.001) and *G. pallidipes* (p < 0.001). The prevalence of *S. glossinidius* in *G. m. morsitans* was significantly higher than in *G. pallidipes* (p < 0.001). A significant difference (p = 0.032) was found between male and female *G. pallidipes* in relation to *S. glossinidius* infection, with males (11.1% 95% CI 1.4-34.7%) having a higher prevalence than females (1.2% 95% CI 0.4-.2.9%). No difference (p = 0.566) was found between male (12.0% 95% CI 2.5-31.2%) and female *G. m. morsitans* (18.8% 95% CI 12.0-.27.2%). Only female *G. brevipalpis* were caught so it was not possible to compare them to male flies.

### Prevalence of trypanosomes in individual species of tsetse

**Figure 2:**
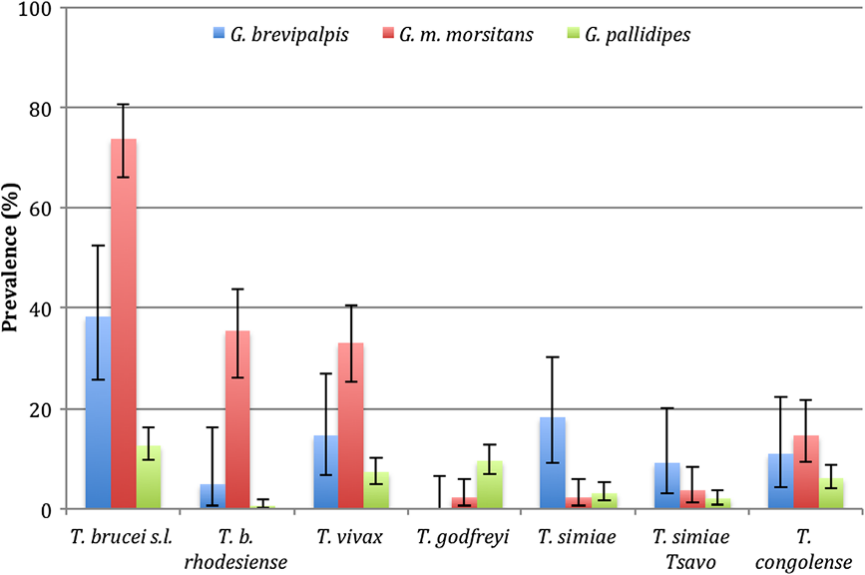
**Prevalence of African trypanosomes detected by ITS-PCR (** ***T. brucei*** **s.l.,** ***T. vivax*** **,** ***T. godfreyi*** **,** ***T. simiae*** **and** ***T. congolense*** **) and SRA-PCR (** ***T. b. rhodesiense*** **) in** ***G. brevipalpis*** **,** ***G. m. morsitans*** **and** ***G. pallidipes*** **from Luambe National Park, Zambia.** Error bars show 95% confidence intervals.

The prevalence of trypanosomes detected by PCR is summarised in Table 1 and illustrated in Figure 2.

**Table 1:** **Prevalence of African trypanosomes detected in Luambe National Park**

Tsetse species	Trypanosome prevalence (95% CI)						
	***T. brucei***s.l.	***T. b. rhodesiense***	***T. vivax***	***T. godfreyi***	***T. simiae***	***T. simiae Tsavo***	***T. congolense***
*Glossina brevipalpis* (n = 55, 42)	38.2% (25.4-52.3)	4.8% (0.6-16.2)	14.5% (6.5-26.7)	0% (0.0-6.5)	18.2% (9.1-30.1)	9.1% (3.0-20.0)	10.9% (4.1-22.2)
*Glossina morsitans morsitans* (n = 137, 119)	73.7% (65.9-80.6)	35.3% (26.0-43.7)	32.9% (25.1-40.3)	2.2% (0.6-5.8)	2.2% (0.6-5.8)	3.6% (1.2-8.3)	14.6% (9.2-21.6)
*Glossina pallidipes* (n = 419, 392)	12.6% (9.6-16.2)	0.5% (0.06-1.8)	7.2% (4.8-10.1)	9.5% (6.9-12.7)	3.1% (1.6-5.3)	1.9% (0.8-3.7)	6.0% (3.9-8.7)

ITS-PCR (*T. brucei* s.l., *T. vivax*, *T. godfreyi*, *T. simiae* and *T. congolense*) and SRA-PLC-PCR (*T. b. rhodesiense*) were used to detect trypanosomes in *G. brevipalpis*, *G. m. morsitans* and *G. pallidipes* from Luambe National Park, Zambia. Number of flies examined has been provided for ITS-PCR and then SRA-PLC-PCR. The latter is lower as flies not positive for PLC were removed from this dataset.

#### G. brevipalpis

Of the 55 flies examined, 56.4% (95% CI 42.3%-69.8%) were positive for trypanosome DNA. Flies that were *T. brucei* s.l. positive were further evaluated to examine if they contained the SRA gene. Of the 21 *G. brevipalpis* (38.2%) that were positive, two were shown to be *T. b. rhodesiense,* six flies were PLC positive, the remaining 13 flies showed no amplification, suggesting that there was not enough DNA present to make a definitive diagnosis. Discounting the 13 flies that did not amplify, this gives an overall prevalence for *T. b. rhodesiense* of 4.8% (95% CI 0.6-16.2%).

DNA from a single species of trypanosome (51.6%) was found in the majority of positive flies. All the flies where two species of trypanosomes were detected (38.7%) involved *T. brucei* s.l. in partnership with *T. simiae*, *T. simiae* Tsavo, *T. congolense* and *T. vivax*. There were two flies (6.5%) where DNA from three trypanosome species was amplified, both involved *T. brucei* s.l. and *T. vivax* with one fly being positive for *T. simiae* and the other being positive for *T. simiae* Tsavo. In one fly (3.2%) four trypanosome species were detected (*T. b. brucei* s.l., *T. congolense*, *T simiae* Tsavo and *T. vivax*).

#### G. m. morsitans

Of the 137 flies examined, 83.9% (95% CI 76.7%-89.7%) were positive for trypanosome DNA. Flies that were *T. brucei* s.l. positive were further evaluated to see if they contained the SRA gene. Of the 101 *G. m. morsitans* (73.7%) that were *T. brucei* s.l. positive, 42 were shown to be *T. b. rhodesiense*, 41 flies were PLC positive while the remaining 18 flies showed no amplification (n = 10) or were VSG positive (n = 8). Discounting these 18 flies, this gives an overall prevalence for *T. b. rhodesiense* of 35.3% (95% CI 26.0-43.7%).

DNA from a single species of trypanosome (53.9%) was found in the majority of positive flies. With the exception of two flies where *T. congolense* and *T. vivax* was detected, all flies with two species of trypanosomes detected (38.3%) involved *T. brucei* s.l. in partnership with *T. vivax*, *T. congolense, T. godfreyi T. simiae* Tsavo or *T. simiae*. There were nine flies (7.8%) where DNA from three trypanosome species was amplified, all of which were positive for *T. vivax*, *T. brucei* s.l. and *T. congolense* DNA.

#### G. pallidipes

Of the 419 flies examined, 29.1% (95% CI 24.8%-33.7%) were positive for trypanosome DNA. Of the 53 *G. pallidipes* that were *T. brucei* s.l. positive (12.6%), two were shown to be *T. b. rhodesiense*, 24 flies were PLC positive while the remaining 27 flies showed no amplification (n = 25) or were VSG positive (n = 2). Discounting these 27 flies, this gives an overall prevalence for *T. b. rhodesiense* of 0.5% (95% CI 0.06-1.8%).

DNA from a single species of trypanosome (68.9%) was found in the majority of positive flies. However, DNA was amplified from two trypanosome species in thirty samples (24.6%). In the majority of these cases this involved *T. brucei* s.l. in partnership with *T. godfreyi*, *T. congolense*, *T. vivax* and *T. simiae*. Other flies in which two species of trypanosome were amplified included *T. godfreyi* in partnership with *T. congolense*, *T. vivax*, *T. simiae* and *T. simiae* Tsavo, there were also flies positive for *T. vivax* and *T. simiae* Tsavo, *T. vivax* and *T. congolense* and *T. congolense* and *T. simiae*. There were seven flies (5.4%) where DNA from three trypanosome species was amplified, the majority of these flies amplified *T. brucei* s.l. and *T. godfreyi* in partnership with *T. simiae*, *T. congolense*, *T. simiae* Tsavo and *T. vivax*. The two other flies, where three species of trypanosomes were amplified, contained *T. vivax*, *T. brucei* s.l. and *T. congolense* and *T. vivax*, *T. godfreyi* and *T. simiae*. There was one fly where four trypanosome species were amplified. This fly contained DNA from *T. vivax, T. godfreyi T. simiae* and *T.simiae* Tsavo.

### Comparison of trypanosomes found in tsetse

*T. brucei* s.l. DNA was identified significantly more frequently in *G. m. morsitans* (73.7%) when compared to *G. brevipalpis* (38.1%, p < 0.001) and *G. pallidipes* (12.6%, p < 0.001). The difference between *G. brevipalpis* and *G. pallidipes* was also significant (p < 0.001). *T. b. rhodesiense* DNA was identified significantly more frequently in *G. m. morsitans* (35.3%) when compared to *G. brevipalpis* (4.8%, p < 0.001) and *G. pallidipes* (0.5%, p < 0.001). The difference between *G. brevipalpis* and *G. pallidipes* was also significant (p = 0.026).

*T. vivax* DNA was amplified significantly more frequently in *G. m. morsitans* (32.8%) when compared to *G. brevipalpis* (14.5%, p = 0.012) and *G. pallidipes* (12.6%, p < 0.001). The difference between *G. brevipalpis* and *G. pallidipes* was not significant (p = 0.066).

*T. godfreyi* DNA was identified significantly more frequently in *G. pallidipes* (12.6%) when compared to *G. brevipalpis* (0%, p = 0.009) and *G. m. morsitans* (2.2%, p = 0.003). The difference between *G. brevipalpis* and *G. m. morsitans* was not significant (p = 0.559).

*T. simiae* DNA was identified in significantly more samples from *G. brevipalpis* (18.2%) when compared to *G. m. morsitans* (2.2%%, p < 0.001) and *G. pallidipes* (3.1%, p < 0.001). The difference between *G. m. morsitans* and *G. pallidipes* was not significant (p = 0.772).

*T. simiae* Tsavo DNA was identified in significantly more samples from *G. brevipalpis* (12.6%) when compared to *G. pallidipes* (1.9%, p = 0.009). There was no significant difference between *G. brevipalpis* and *G. m. morsitans* (3.6%, p = 0.153) and *G. m. morsitans* and *G. pallidipes* (p = 0.195).

*T. congolense* DNA was found significantly more frequently in *G. m. morsitans* (14.6%) when compared to *G. pallidipes* (6.0%, p = 0.003). There was no significant difference between *G. m. morsitans* and *G. brevipalpis* (10.9%, p = 0.642) and *G. brevipalpis* and *G. pallidipes* (p = 0.155).

With the exception of *T. simiae Tsavo* in *G. m. morsitans* where males were more likely to be positive than females there was no difference between trypanosome infection and gender (data not shown).

### Association between *S. glossinidius*and trypanosome infection

No association was found between the presence of *S. glossinidius* and trypanosomes that undergo development in the midgut in *G. brevipalpis* (p = 1), *G. m. morsitans* (p = 0.103) or *G. pallidipes* (p = 0.686). There was also no association detected at the individual trypanosome species level for any of the fly species examined (data not shown).

## Discussion

### *S. glossinidius*prevalence

Significant differences were detected between the three species of tsetse examined in the current work. Almost all the *G. brevipalpis* flies were infected with *S. glossinidius* while very few *G. pallidipes* were infected. There are very few publications on *S. glossinidius* prevalence in wild tsetse, however, the results obtained for *G. brevipalpis* are similar to those from Tanzania where 100% (95% CI 80.5-100%) of flies were found to be infected [31]. For *G. m. morsitans* the prevalence is similar to that obtained for flies in Zimbabwe where 28.5% (95% CI 19.6-39%) of flies were positive [31]. For *G. pallidipes* the prevalence of *S. glossinidius* is much lower than that obtained in Kenya [23], Tanzania and Zimbabwe [31] where rates of infection were 15.9% (95% CI: 12–20.5%), 83.3% (95% CI 74–90.4%) and 17.6% (95% CI 11.3-25.7%) respectively. Studies on other species of tsetse have shown varying rates of infection, for example Farikou *et al*. [22] reported a prevalence of 55% in *G. p. palpalis* in Cameroon, while no *S. glossinidius* were found in *Glossina fuscipes fuscipes* from Kenya [34] or Uganda [35].

Although in the current work we found a significant difference between genders in terms of *S. glossinidius* infection in *G. pallidipes*, this was most likely due to the low numbers of male flies caught. Higher levels of *S. glossinidius* were reported in female *G. austeni* by Wamwiri *et al*. [23], however, there was no difference between genders for *G. pallidipes*. Matthew [31] found no significant difference between genders for *S. glossinidius* infection in *G. m. morsitans* and *G. pallidipes* from Zimbabwe and *G. pallidipes* from Tanzania.

### Trypanosome detection in tsetse flies

A wide variety of trypanosomes were detected in the three species of tsetse sampled in the current work. We were surprised to find such a high *T. brucei* s.l. prevalence within *G. m. morsitans*, this could suggest that they are quite susceptible to infection by these trypanosomes or could be a reflection of tsetse feeding habits. As DNA was extracted from the whole fly it is impossible to tell if the infections had matured, if they represented immature infections only or were dying trypanosomes from a residual bloodmeal. During collection of the tsetse used in this study, Anderson [27] undertook dissection studies to estimate infection rates in *G. pallidipes* and *G. m. morsitans*. Results showed that very few flies had mature infections and no *T. brucei* s.l. species were detected during the study. Of the 1293 *G. pallidipes* dissected, the prevalence of *Duttonella* species was 2.86% (95% CI 2.02-3.92) and *Nannomonas* species was 1.31% (95% CI 0.77-2.10). A further 266 *G. m. morsitans* flies were dissected with a *Duttonella* prevalence of 4.89% (95% CI 2.63-8.21) and *Nannomonas* prevalence of 3.38% (95% CI 1.56-6.33). A total of eight immature infections were detected in *G. pallidipes* and four in *G. m. morsitans* and these could have been either *Nannonomas* or *T. brucei* s.l. Even if all these infections had been *T. brucei* s.l. infections, the infection rates would only be 0.62% (95% CI 0.27-1.22) and 1.50% (95% CI 0.41-3.81), which are 20 and 49 fold lower than that diagnosed by PCR. Although PCR is more sensitive than microscopical techniques, it is unclear in this case if the results presented here are actual infections or just the PCR amplifying trypanosome DNA from a recent bloodmeal that will ultimately not lead to an active midgut infection. Given that Anderson [27] found very few active infections in tsetse during dissection studies in the study area this might suggest that the high prevalence is a result of high prevalence in the local wildlife. Similar results were obtained by Malele *et al*. [36], with dissection studies showing low levels of infection (*T. brucei* s.l. = 2.5%) and PCR showing much higher levels of infection (*T. brucei* s.l*.* = 55%), a 22 fold difference. Therefore, further work might be required in order to verify the use of PCR to diagnose trypanosome infections in wild tsetse.

The variation between species might be due to tsetse host preference. Although no bloodmeal analysis was carried out on the current samples, previous work had shown that *G. brevipalpis* normally feed on hippopotamus [37]. *G. m. morsitans* normally feed on Suidae (mostly warthog and bushpig), hippopotamus and ruminants (cattle, bushbuck, buffalo and other wild ruminants). Samples from the Luangwa Valley, Zambia, showed these tsetse primarily fed on vervet monkey, baboon, man, side-striped jackal, domestic dog, lion, elephant, black rhinoceros, Burchell’s zebra, bushpig, warthog, hippopotamus, giraffe, buffalo, bushbuck, kudu, duiker, waterbuck, roan antelope, hartebeest, impala and domestic ox [37]. However, there are no domesticated animals found in this part of the Luangwa Valley, due largely to the high trypanosomiasis challenge. *G. pallidipes* normally feed on ruminants and Suidae, however, although samples from Zambia were analysed no specific information on them is provided [37]. The high prevalence of *T. b. rhodesiense* in *G. m. morsitans* was surprising and further supports different feeding habits by the three tsetse species sampled in the current work. Work by Anderson *et al*. [33] found that the majority of *T. brucei* s.l. infections in wildlife were concentrated in four species (bushbuck, waterbuck, lion and leopard). *T. b. rhodesiense* was identified in wildlife from the nearby game management areas of Musalangu and Lower Lupande. The overall prevalence for *T. b. rhodesiense* was 0.5% (95% CI 0.06–1.72%) in the Luangwa Valley with the animals positive for *T. b. rhodesiense* being a bushbuck and a buffalo. Also of interest in this study is the low detection rate of *T. godfreyi* in *G. m. morsitans* in comparison with *G. pallidipes.* As the warthog is considered the definitive host for this parasite [38] and *G. m. morsitans* are believed to feed predominantly on suidae it may suggest a wider host range for this parasite. Bloodmeal analysis on the tsetse flies analysed in the current work may shed some light on what the three species of tsetse feed on and why the rates vary widely between tsetse species.

There have been very few papers where tsetse flies have been investigated for *T. b. rhodesiense*. Auty *et al*. [11] analysed 133 samples by PCR that were positive for trypanosomes by microscopy, the overall prevalence of *T. brucei* s.l. was 0.8% (95% CI: 0.05-1.2%) and 0.7% (95% CI: 0.4-1.2%) for *Glossina swynnertoni* and *G. pallidipes* respectively. Of the *T. brucei* s.l. positive flies, around 10% of flies were positive for SRA, giving an overall prevalence of 0.01% (95% CI: 0–0.54) for *G. swynnertoni* and 0.0085% (95% CI 0–0.059) for *G. pallidipes*. Further work in Tanzania found similar levels of *T. brucei* s.l. positive *G. swynnertoni* (0.7%, 95% CI 0.2-1.7%), however, in this case all were also positive for SRA [39]. These results are much lower than what we found in the current work in Zambia and might be related to trypanosomes found in mammals in the area where the flies were captured. In the current work the proportion of PLC positive to *T. b. rhodesiense* in terms of *G. brevipalpis* was 0.25 (2 from 8), for *G. m. morsitans* it was 0.51 (42 from 83) and 0.08 (2 from 24) for *G. pallidipes*. Although it is not possible to tell if flies that were positive for *T. b. rhodesiense* were also positive for *T. b. brucei* the ratio of infection for the 3 fly species was 0.3, 1.02 and 0.09 for *G. brevipalpis*, *G. m. morsitans* and *G. pallidipes* respectively. There is little published work looking at *T. b. rhodesiense* in Zambia, however, recently Lisulo *et al*. [40] and Namangala *et al*. [41] have found human infective parasites in dogs in the Luangwa valley and there have been reports of both tourists [42, 43] and locals being infected with *T. b. rhodesiense* [44], suggesting there is active transmission in the area.

**Figure 3:**
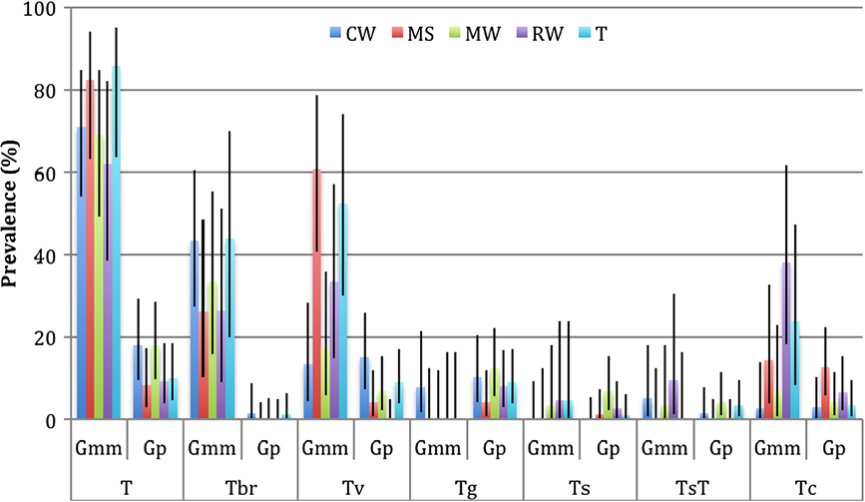
**Bar chart showing the distribution of trypanosomes in** ***G. m. morsitans*** **and** ***G. pallidipes*** **by vegetation type.** Key: CW, *Combretum-Terminalia* woodland; MS, mopane scrub woodland; MW, mopane woodland; RW, riverine woodland; T, thicket; Gmm, *G. m. morsitans*; Gp, *G. pallidipes*; T, *T. brucei* s.l.; Tbr, *T. b. rhodesiense*; Tv, *T. vivax*; Tg, *T. godfreyi*; Ts, *T. simiae*; TsT, *T. simiae*; Tc, *T. congolense*. Error bars show 95% confidence intervals.

When the distribution of trypanosome infections is considered by vegetation type, no clear pattern emerges. Although discreet vegetation units occur in Luambe National Park, the mobility of both hosts and vectors means that variation in infection rates due to habitat influences may not be evident at this scale. However, of interest in this study is the high infection rate with *T. b. rhodesiense* in *G. m. morsitans* in both *Combretum-Terminalia* woodland and thicket (Figure 3).

### Association between *S. glossinidius*and trypanosome infection

In the current work no association was detected between the presence of *S. glossinidius* and presence of trypanosomes in any of the three tsetse species examined. This is in disagreement with recent work published by Farikou *et al*. [22] and Wamwiri *et al*. [23] for *G. p. palpalis* and *G. pallidipes* respectively, but does agree with work undertaken on *G. austeni* [23]. In terms of *G. pallidipes* this could be due to the low prevalence of *S. glossinidius* detected in the current work, with the exception of *T. godfreyi*, *G. pallidipes* in many cases had significantly fewer trypanosomes present than *G. m morsitans* and *G. brevipalpis*. However, almost all *G. brevipalpis* flies (92.7%) were infected with *S. glossinidius*, despite this, this species of tsetse did not contain the most trypanosome positive flies (50.9% of flies with a trypanosome infection that establishes in the midgut). Instead it was *G. m. morsitans* where the prevalence of *S. glossinidius* was 17.5%, where the most trypanosome infected flies was observed (78.1% with midgut trypanosomes). As described above, a number of reasons could explain the detection of trypanosomes in these samples. For example host preference and detection of DNA from residual bloodmeals. Tsetse susceptibility has also been linked to the teneral phenomenon, where tsetse are most susceptible to infection on their first feed [45], as such if a susceptible fly does feed on an uninfected host, it will therefore become refractory to infection at future bloodmeals.

Molecular methods of detection are more sensitive than traditional dissection techniques and enable the assessment of mixed infections, but for various reasons it may be difficult to assess an association between *S. glossinidius* and trypanosome infection on field collected tsetse, particularly when flies are analysed only by molecular methods. Although the study by Farikou *et al*. [22] combined both techniques, this approach also has limitations. For example, trypanosome-susceptible flies that fed on an animal not infected with trypanosomes would become refractory to later infection (as susceptibility to infection is largely a teneral phenomenon [45]). In contrast, if the same fly had fed on a trypanosome positive animal it would have become infected, so despite being a susceptible fly, using their approach it would be classified as a refractory fly. Although in the current work we could not discriminate between established infections in the tsetse and residual bloodmeal contamination, one way to investigate this further would be to trap live flies and then feed them on a clean bloodmeal source for period of at least a week. This would allow any residual DNA to be cleared from the fly meaning that only established infections would be present. However, as mentioned above, this would still allow susceptible flies that have become refractory to infection by feeding on an uninfected host to be included. When investigating establishment of trypanosome infections in tsetse, Harley [46, 47] showed that the majority of the tsetse emerging from pupae collected from the wild, which were then fed on infected rats were refractory to infection. Therefore, the best method to investigate if *S. glossinidius* does have an impact on tsetse susceptibility would be to collect tsetse pupae from the wild and examine flies for trypanosomes and *S. glossinidius* after an infective bloodmeal was allowed to establish in the tsetse. This would be the only way to control for both susceptible flies becoming refractory following a feed on a non-infected animal and residual blood contamination through feeding on infected hosts.

## Conclusion

The current work shows that prevalence of *S. glossinidius* varies within species of tsetse caught within the same area, and that these tsetse were positive for a variety of trypanosomes. Whether or not this is linked to the susceptibility of these tsetse flies to certain trypanosome species, or is a function of host preference is unclear. The high trypanosome prevalence (particularly *T. brucei* s.l. in *G. m. morsitans*) detected by PCR in the current work but low prevalence detected by microscopy [27] might suggest a high level of infection in the local wildlife, however, the fly population might be highly refractory to infection.

Finally, no link was found between presence of *S. glossinidius* and presence of trypanosome DNA; flies were diagnosed trypanosome positive by PCR rather than by microscopy and therefore this might wrongly diagnose a fly as trypanosome positive due to it being recently fed. The discrepancy between dissection results and PCR analysis requires further analysis and therefore trying to associate *S. glossinidius* presence and trypanosome susceptibility should be interpreted with caution.
